# Facteurs associés à l'acceptabilité du paiement numérique mobile chez les agents de santé du district sanitaire de Koumpentoum Sénégal en 2023

**DOI:** 10.11604/pamj.2024.49.32.44475

**Published:** 2024-10-11

**Authors:** El Hadji Cheikh Abdoulaye Diop, Amadou Ibra Diallo, Adélaïde Ndew Dog, Ibrahima Ndiaye, Bayal Cissé, Souleymane Ndiaye, Mouhamadou Faly Ba, Adama Faye

**Affiliations:** 1 District Sanitaire de Tambacounda, Ministère de la Santé et de l'Action Sociale, Dakar, Sénégal,; 2 Institut de la Santé et du Développement, Université Cheikh Anta DIOP de Dakar, Dakar, Sénégal,; 3 Institut Supérieur de Management, Campus de Kaolack, Kaolack, Sénégal,; 4 Direction Régionale de la Santé Tambacounda, Ministère de la Santé et de l'Action Sociale, Dakar, Sénégal,; 5 Faculté des Sciences Économiques et Sociales, Université Iba Der Thiam de Thiès, Thiès, Sénégal

**Keywords:** Acceptabilité, paiement, mobile, santé, Sénégal, Acceptability, payment, mobile, health, Senegal

## Abstract

**Introduction:**

la numérisation du paiement des agents de santé pourrait améliorer leur bien-être, celui des usagers des points de prestations sanitaires et les performances du système de santé. L'objectif de cette étude était d'identifier les facteurs associés à l'acceptabilité du paiement mobile chez les agents de santé du district sanitaire de Koumpentoum.

**Méthodes:**

il s'agissait d'une étude transversale descriptive et à visée analytique, réalisée en janvier 2023 au niveau du district sanitaire de Koumpentoum situé à l'est du Sénégal. Le recrutement était exhaustif auprès de l'ensemble du personnel de santé et des acteurs communautaires. Un questionnaire a été élaboré et administré à distance par appel téléphonique. Il avait permis de recueillir des informations sur les caractéristiques socio-professionnelles, les connaissances, attitudes, pratiques ainsi que sur l'acceptabilité du paiement mobile par les agents de santé. Une régression logistique binomiale a été utilisée pour identifier les facteurs associés à l'acceptabilité du paiement mobile par les agents de santé.

**Résultats:**

au total, 418 agents ont participé à l’enquête, parmi lesquels 87,3% étaient des acteurs communautaires. L'ensemble des enquêtés disait que le paiement numérique dans leur contexte était représenté par les transferts via les applications mobiles et 86,6% étaient favorable à son utilisation dans le domaine de la santé. La majorité (91,5%) accepteraient d'être payé par ce mode. Les motifs d'adhésion étaient les bonnes perceptions sur la commodité, la rapidité et la sécurité et les motifs de non-adhésion étaient le manque de réseau, les erreurs et les frais de transfert. Les facteurs favorisants l'acceptabilité du paiement mobile en santé étaient une expérience professionnelle inférieure à 5 ans (ORa= 7,91 [2,65-34,38]; p= 0,001), une expérience satisfaisante du paiement numérique mobile (ORa= 4,18 [1,3-18,9]; p= 0,031), l'acceptabilité du paiement mobile dans la vie quotidienne (ORa= 5,81 [1,37-23,29]; p= 0,013) et l'attente de performances (ORa= 7,3 [3,42-16,05]; p < 0,001).

**Conclusion:**

cette étude menée dans le district sanitaire de Koumpentoum indiquait une forte acceptation du paiement mobile parmi les agents de santé. Malgré cela, des défis potentiels comme le manque de réseau et les frais de transfert sont relevés. Bien que l'intégration du paiement mobile dans le secteur de la santé à Koumpentoum semble prometteuse, des mesures sont nécessaires pour surmonter ces obstacles identifiés.

## Abstract (en)

**Introduction:**

digitising health worker payments could improve their well-being, that of users of health service points and the performance of the health system. The purpose of this study was to identify factors associated with the acceptability of mobile payments among health workers in the Koumpentoum health district.

**Methods:**

we conducted a cross-sectional, descriptive and analytical study in the Koumpentoum health district, in eastern Senegal, in January 2023. Recruitment was exhaustive, involving all health staff and community stakeholders. A questionnaire was developed and administered remotely via phone calls. It was used to gather information on socio-professional characteristics, knowledge, attitudes, practices and the acceptability of mobile payments by healthcare workers. Binomial logistic regression was used to identify factors associated with the acceptability of mobile payments by health workers.

**Results:**

a total of 418 health workers were surveyed, 87.3% of whom were community actors. All respondents said that digital payments in their context were represented by transfers via mobile applications, and 86.6% were in favor of its use in the healthcare sector. The majority of them (91.5%) would accept being paid through this method. Reasons for adoption included positive perceptions of convenience, speed and security, while reasons for non-adoption were lack of network access, errors and transfer fees. Factors favoring the acceptability of mobile healthcare payments included less than 5 years of professional experience (ORa= 7.91 [2.65-34.38]; p= 0.001), a satisfactory experience with mobile digital payments (ORa= 4.18 [1.3-18.92]; p= 0.031), acceptability of mobile payments in daily life (ORa= 5.81 [1.37-23.29]; p= 0.013) and performance expectations (ORa= 7.3 [3.42-16.05]; p < 0.001).

**Conclusion:**

this study conducted in the Koumpentoum health district indicated a high level of acceptance of mobile payments among health workers. Despite this, potential challenges such as lack of network access and transfer fees were noted. While the integration of mobile payments in the health sector in Koumpentoum appears promising, measures are needed to overcome these identified obstacles.

## Introduction

Il n'existe pas de définition unique du paiement numérique appelé aussi paiement électronique ou encore paiement en ligne. Les paiements électroniques peuvent être totalement, principalement ou partiellement numériques. Le paiement numérique peut être considéré comme un échange d'argent par système électronique. Il s'agit des paiements réalisés sur Internet ou via des réseaux de télécommunications, générés soit à partir d'un ordinateur ou d'un téléphone mobile [1,2]. La pandémie COVID-19 a accéléré les efforts de mise en l'échelle du paiement numérique dans tous les secteurs de la vie économique notamment dans le domaine de la santé et même dans les pays en développement [3]. Les avantages perçus du paiement numérique étaient cependant déterminés par la confiance et les caractéristiques sociales correspondant au contexte de la pandémie [4]. La numérisation des motivations financières des prestataires pourrait améliorer le bien-être des agents de santé, les performances des systèmes de santé et le bien être des patients. Cependant, les évidences dans le domaine du paiement numérique en santé sont rares [5,6]. Si la digitalisation des paiements est effective en Europe où 80% des entreprises utilisent le paiement numérique [7], en Afrique notamment au Sénégal 80% des travailleurs reçoivent encore leurs salaires en espèces. La digitalisation de la paie transforme la vie économique, sociale et financière des travailleurs [8].

Les études portant sur les facteurs associés à l'acceptabilité du paiement numérique dans le domaine de la santé sont rares. Le Rajasthan en Inde a été le premier Etat au monde à mettre en place un système de paiement numérique des travailleurs de santé de 2015 à 2020 avec comme résultants une augmentation de la motivation financière des prestataires et des performances de programmes de santé [9]. Les auteurs rapportent dans les études réalisées dans d'autres secteurs, une association entre l'acceptabilité du paiement numérique, la valeur du prix, la confiance, les perceptions de traçabilité, de commodité, de sécurité, d'anonymat, de facilité, de vitesse, l'espérance hédonique et l'attente de performances [10,11]. L'utilisation informelle du paiement numérique dans le secteur de la santé au Sénégal suggère une tendance à l'adoption de méthodes de transaction électronique en dehors des canaux officiels. Cette pratique peut être le résultat de la rapidité et de la commodité perçues offertes par le paiement numérique [10,11]. Cependant, elle souligne également la nécessité de régulariser et de formaliser ces transactions pour garantir la sécurité, la transparence et l'efficacité du système de paiement dans le secteur de la santé. Des études approfondies pour comprendre les motivations et les défis associés à cette utilisation informelle pourraient aider à orienter des politiques visant à intégrer le paiement numérique de manière officielle et optimale dans le domaine de la santé au Sénégal [12]. A ce jour aucune enquête sur le paiement numérique en santé n'a été menée dans le pays à notre connaissance. Ce qui dénote l'originalité de cette étude visant à augmenter la masse critique d'évidences scientifiques en vue d’aider à la prise de décision éclairée pour l'implémentation d'un système de paiement numérique des agents de santé. L'objectif de cette étude était d'identifier les facteurs associés à l'acceptabilité du paiement mobile par les agents de santé du district de Koumpentoum.

## Méthodes

**Cadre d'étude:** le district sanitaire de Koumpentoum, correspondant au département du même nom est situé dans la région de Tambacounda à l'est du Sénégal. Il est situé à 385 Km de la capitale Dakar. En 2022, sa population s'élevait à 176 666 habitants avec une densité de 23 habitants par km^2^. Le district comptait 23 points de prestation sanitaire, répartis en 22 postes de santé et 1 centre de santé de référence. Plus de la moitié de la population, soit 51%, vivait à moins de 5 km d'un point de prestation sanitaire (zone fixe et fixe déplacée), 35% se situait entre 5 et 15 km (zone avancée), et 14% résidait à plus de 15 km (zone mobile) [13]. En 2022, on dénombrait 1204 agents de santé composés de 61 agents qualifiés, 86 agents non qualifiés, 230 acteurs communautaires de soins et 827 acteurs communautaires de prévention et de promotion. L'utilisation du téléphone était très élevée avec 75% mais la couverture du réseau téléphonique faisait défaut dans les zones rurales. Les points de vente de paiement mobile étaient disponibles dans tous les villages abritant les points de prestations sanitaires [14].

**Type et période d'étude:** il s'agissait d'une étude transversale, descriptive et à visée analytique qui s'est déroulée durant le mois de janvier 2023 au niveau des aires géographiques des 23 structures du district sanitaire de Koumpentoum.

**Population d'étude:** la population d'étude était constituée des agents de santé du district sanitaire de Koumpentoum. Elle était composée d'agents qualifiés incluant les médecins, les techniciens supérieurs, les sages-femmes, les infirmiers et les assistants infirmiers et non qualifiés constitués par les acteurs communautaires de soins, les matrones, les relais et les Bagénu Gox (marraines de la santé).

**Recrutement**: nous avons procédé au recrutement exhaustif des agents de santé du district sanitaire de Koumpentoum.

**Collection des données:** nous avons collecté les contacts téléphoniques des agents auprès du point focal ressources humaines. Un questionnaire a été élaboré à partir des différents items du cadre conceptuel de l'acceptabilité de l'innovation d'une technologie en santé [15]. Il a été administré par des entretiens téléphoniques et comportait des items sur les connaissances, attitudes, pratiques, satisfaction et l'acceptabilité du paiement mobile. Nous avons recruté et formé en 5 jours 10 enquêteurs et 2 superviseurs. Un pré-test du questionnaire a été réalisé auprès des 20 agents du district voisin de Maka Colibantang. Le questionnaire a été secondairement consolidé avant l'enquête proprement dite.

**Données collectées:** la variable dépendante était l'acceptabilité du paiement mobile en santé, c'est-à-dire la disposition à recevoir sa rémunération de manière numérique à travers son téléphone portable. Les variables indépendantes ont été conceptualisées selon le modèle de la théorie unifiée de l'acceptation de l'utilisation des technologies (TUAUT2), tel qu'adapté par Al-Okaily *et al*. [15] dans leur étude sur les déterminants de l'acceptation des systèmes de paiement numériques en vertu des différences d'orientation culturelle avec le cas de l'évitement de l'incertitude. Elles portent sur les éléments suivants:

**Condition facilitante:** le revenu mensuel, le niveau d'instruction, la catégorie socioprofessionnelle, la durée d'expérience professionnelle, la préférence pour le paiement mobile, la possession de téléphone, le type de téléphone, l'utilisation, la fréquence, les montants utilisés et les applications du paiement numérique ont été les conditions facilitantes.

**Niveau de connaissance sur le paiement numérique en santé:** la connaissance du paiement numérique comportait 6 items du paiement numérique à savoir la connaissance de sa définition, des opérateurs disponibles, des services offerts, des tarifs, de son mode d'utilisation et de la possibilité de son utilisation par les programmes de santé. Le score variait 0 à 6. On considérait qu'une personne a une connaissance complète lorsqu'elle avait le score maximum.

### Analyse des données

Le logiciel R version 4.2.2. avait été utilisé pour analyser les données. Les variables quantitatives ont été décrites en analyse descriptive en utilisant la médiane, les extrêmes, la moyenne avec son écart type alors que les variables qualitatives ont été décrites en utilisant les fréquences absolues et relatives. Dans l'analyse bivariée, les tests statistiques Chi^2^ de Pearson et le test de Fisher avaient permis de vérifier l'existence de lien statistique entre les variables indépendantes et l'acceptabilité du paiement mobile. En analyse multivariée, nous avions utilisé une régression logistique binomiale qui avait inclus toutes les variables indépendantes avec un p < 0,25 retrouvé dans l'analyse bivariée et celles dont la revue de la littérature avait retrouvé un lien avec l'acceptabilité du paiement mobile. La méthode pas à pas descendante avait permis de retenir les variables associées à l'acceptabilité du paiement mobile au seuil p < 5%. Secondairement, ces variables ont été retirées une à une avec une comparaison des modèles emboîtées par Aikake Information Criterion (AIC) [16]. La démarche a été poursuivie jusqu'à ce qu'aucune amélioration ne soit retrouvée par le test du maximum de vraisemblance. Le test d'Hosmer-Lemeshow [17] avait permis de vérifier l'adéquation du modèle final. La force des liens entre l'acceptabilité du paiement mobile et les variables indépendantes sera appréciée en bivariée avec l'OR et en multivariée avec l'OR ajusté, entourées de leurs intervalles de confiance à 95% (IC 95%).

### Considérations éthiques

L'étude avait obtenu un avis technique et scientifique favorable du Comité National de la Recherche Scientifique (CNERS) par la note de service N°311/MSAS/CNERS/SP du 1^er^ décembre 2022. De plus, l'autorisation administrative pour mener des recherches pendant une année a été délivrée par la Direction de la Planification, de la Recherche et des Statistiques (DPRS) par la note de service N°1531/MSAS/DPRS/DR le 2 décembre 2022. Avant leur participation à l'étude, les agents de santé ont été informés de l'enquête. Un consentement libre et éclairé a été recueilli auprès de chaque participant de l'étude. Les données collectées ont été traitées et conservées dans manière anonyme et confidentielle. Il n'existait pas de rémunération pour les participants qui pouvaient se retirer de l'entretien téléphonique sans aucune contrainte.

## Résultats

### Caractéristiques sociodémographiques et professionnelles

On observait une proportion d'hommes de 54,07%, soit un sexe ratio hommes/femmes de 1,083. L'âge moyen était de 37,5 ans, avec un écart type de 9,85 ans. L'âge médian était 37 ans, avec un minimum de 18 ans et un maximum de 65 ans. Les personnes de plus de 35 ans représentaient 59,57% de l'échantillon. La proportion de personnes mariées était de 87,8%, et 61,72% des agents avaient un revenu mensuel inférieur à cinquante mille francs CFA. La proportion d'agents de santé ayant un niveau d'instruction moyen secondaire était de 32,3% tandis que 15,79% avaient un niveau d'études supérieur. Les agents de santé qualifiés ne représentaient que 12,68% des effectifs. La durée moyenne de l'expérience était de 8,02 ans avec un écart type de 7,24 ans. La durée d'expérience professionnelle de moins de 5 ans concernait 36,12% des participants (Tableau 1).

**Tableau 1:** répartition des agents de santé selon les caractéristiques sociodémographiques et professionnelles (n=418)

Variables	Modalités	Fréquences absolues (n)	Fréquences relatives (%)
Genre	Masculin	226	54,07
	Féminin	192	45,95
Catégorie d'âge	35 ans et Plus	249	59,57
	Moins de 35 ans	169	40,43
Vie en couple	Oui	367	87,80
	Non	51	12,2
Revenu mensuel < 50 000 FCFA	Oui	258	61,72
	Non	160	38,28
Vie en couple	Oui	367	87,8
	Non	51	12,2
Instruction en français	Oui	267	63,87
	Non	151	36,12
Durée expérience professionnelle	5 ans et plus	267	63,88
	Moins de 5 ans	151	36,12

### Connaissances, attitudes et pratiques sur le paiement numérique en santé

On notait une forte connaissance (94,5%) et une utilisation régulière (77,36%) du paiement mobile dans les activités quotidiennes. Cependant, une majorité (67,23%) ne connaît pas les programmes de santé utilisateurs de ce système de paiement. Tous les participants possèdent un téléphone portable, principalement des smartphones (84,93%). Seulement une minorité (16,27%) a déjà effectué des paiements numériques dans le domaine de la santé (Tableau 2).

**Tableau 2:** répartition des agents de santé selon les connaissances, attitudes et pratiques sur le paiement mobile en santé (n=418)

Variables	Modalités	Fréquences absolues (n)	Fréquences relatives (%)
Connaissance du paiement numérique	Oui	395	94,5
	Non	23	5,5
Connaissance programmes de santé utilisateurs	Oui	137	32,77
	Non	281	67,23
Possession de téléphone portable	Oui	418	100
	Non	0	0
Genre de téléphone portable	Oui	355	84,93
	Non	63	15,07
Utilisation du paiement numérique en routine	Oui	402	77,36
	Non	16	22,64
Déjà payé par voie numérique en santé	Oui	68	16,27
	Non	350	83,73

### Acceptabilité et satisfaction sur le paiement mobile en santé

La proportion d'agents favorable au paiement numérique dans d'autres aspects de la vie quotidienne était de 96,65%. Par contre 86,6% étaient en faveur du paiement numérique dans le secteur de santé, et 85,17% estimaient que les coûts liés au paiement numérique dans le domaine de la santé étaient bas. De plus, 77,36% des agents utilisaient le paiement numérique dans leur routine quotidienne. L'acceptabilité du paiement mobile était très élevée, atteignant 91,5%. Les principales raisons d'adhésion au paiement numérique en santé étaient la perception de la commodité et l'attente de performances, citées par 86,74% des participants, la rapidité pour 70,16%, et la sécurité pour 63,81%. Les raisons de la non-adhésion comprenaient le manque de réseau (92,85%), les erreurs (89,28%), et les frais d'envoi et de transfert (64,28%). Le taux de satisfaction des agents de santé ayant été payés par voie mobile était de 95,58%. Parmi eux, 80,88 ont été très satisfaits et 14,7% satisfaits (Tableau 3).

**Tableau 3:** répartition des agents de santé selon leur acceptabilité et leur satisfaction sur le paiement mobile en santé

Variables	Modalités	Fréquences absolues (n)	Fréquences relatives (%)
Acceptabilité paiement numérique en général	Oui	404	96,65
	Non	14	3,35
Acceptabilité paiement numérique en santé	Oui	362	86,6
	Non	56	13,4
Acceptabilité du paiement mobile en santé	Oui	381	91,5
	Non	37	8,5
Perception de couts d'utilisation faibles	Oui	356	85,17
	Non	62	14,83
Motifs d'adhésion au paiement numérique en santé	Rapidité	254	70,16
	Anonymat	191	52,56
	Facilité	156	43,01
	Traçabilité	195	53,86
	Commodité	314	86,74
	Sécurité	231	63,81
	Performances	314	86,74
Motifs de non adhésion au paiement numérique en santé	Manque de réseau	52	92,85
	Dépense plus	1	1,78
	Erreurs d'utilisation	50	89,28
	Frais d'utilisation	36	64,28
Satisfaction	Oui	65	95,58
	Non	3	4,41
Niveau de satisfaction	Très satisfait	55	80,88
	Satisfait	10	14,7
	Moyennement	3	4,41

### Identification des facteurs associés à l'acceptabilité du paiement mobile

En analyse bivariée, la connaissance des programmes utilisateurs, la perception de sécurité, de rapidité, de coûts d'utilisations faibles, l'attente de performances étaient associés. L'expérience professionnelle de moins de 5 ans, le fait de disposer d'un smartphone et le fait d'être favorable au paiement numérique en général étaient associés à l'acceptabilité du paiement mobile dans le domaine de la santé (Tableau 4). En régression logistique binomiale, une expérience professionnelle inférieure à 5 ans (ORa=7,91 [2,65-34,38]; p= 0,001), une expérience de paiement numérique avec satisfaction (ORa= 4,18 [1,3-18,92]; p= 0,031), l'acceptabilité du paiement numérique dans la vie quotidienne (ORa= 5,81 [1,37-23,29]; p= 0,013) et l'attente de performances (ORa= 7,3 [3,42-16,05]; p <0,001) favorisaient l'acceptabilité du paiement mobile chez les agents de santé (Tableau 5).

**Tableau 4:** identification des facteurs associés à l'acceptabilité du paiement mobile chez les agents de santé en analyse bivariée

Variables	Modalités	Acceptabilité		p
**Oui**	**Non**
Genre	Masculin	208 (49,76%)	18	0,495
	Féminin	173 (42,1%)	19	
Catégorie d'âge	35 ans et plus	221 (52,87%)	28	0,052
	Moins de 35 ans	160 (38,27%)	9	
Vie en couple	Oui	332 (79,42%)	35	0,289
	Non	49 (11,78%)	2	
Instruction en français	Oui	275 (65,78%)	28	0,705
	Non	106 (25,35%)	9	
Catégorie socioprofessionnelle	Diplômés	50 (11,96%)	3	0,603
	Non diplômés	331 (79,18%)	34	
Expérience professionnelle	5 ans et plus	148 (35,40%)	3	<0,001
	Moins de 5 ans	233 (55,74%)	34	
Revenu mensuel	50 milles et plus	232 (55,50%)	26	0,292
	Moins de 50 milles	149 (35,64%)	11	
Genre de téléphone	Smartphone	331 (79,18%)	24	<0,001
	Digital	50 (11,96%)	13	
Utilisation du paiement numérique	Oui	369 (88,27%)	33	0,043
	Non	12 (2,87%%)	4	
Déjà payé par voie numérique en santé	Oui	64 (15,31%)	4	0,484
	Non	317 (75,83%)	33	
Expérience avec satisfaction	Oui	62 (14,83%)	3	0,239
	Non	319 (76,41%)	34	
Favorable au paiement numérique en général	Oui	372 (88,99%)	32	0,004
	Non	9 (2,15%)	5	
Connaissance complète du paiement numérique	Oui	358 (85,64%)	37	0,246
	Non	23 (5,50%)	0	
Connaissance des programmes utilisateurs	Oui	270 (64,59%)	11	<0,001
	Non	111 (25,55%)	26	
Perception de la sécurité du paiement en santé	Oui	221 (52,87%)	10	<0,001
	Non	160 (38,27%)	27	
Perception rapidité paiement numérique santé	Oui	243 (58,13%)	11	<0,001
	Non	138 (33,01%)	26	
Perception facilité paiement numérique santé	Oui	143 (34,21%)	13	0,859
	Non	238 (56,93%)	24	
Attente de performances et commodité	Oui	299 (71,53%)	15	<0,001
	Non	82 (19,61%)	22	
Couts d'utilisation faibles	Oui	333 (79,66%)	23	<0,001
	Non	48 (11,48%)	14	
Perception de l'anonymat du paiement en santé	Oui	176 (42,10%)	15	0,626
	Non	205 (49,04%)	22	
Perception traçabilité paiement numérique santé	Oui	181 (43,30%)	14	0,302
	Non	200 (47,84%)	23	

**Tableau 5:** identification des facteurs associés à l'acceptabilité du paiement mobile chez les agents de santé en analyse multivariée

Variables	Modalités	OR	IC	p value
Expérience professionnelle inférieure à 5 ans	Oui	Référence	[2,65-34,38]	0,001
	Non	7,91		
Expérience de paiement numérique avec satisfaction	Oui	Référence	[1,3-18,92]	0,031
	Non	4,18		
Acceptabilité du paiement numérique au quotidien	Oui	Référence	[1,37-23,29]	0,013
	Non	5,81		
Attente de performances	Oui	Référence	[3,42-16,05]	<0,001
	Non	7,4		

## Discussion

Les résultats de notre étude révèlent une prédominance masculine, avec 54,07% d'hommes et une majorité de personnes mariées, représentant 87,8% de l'échantillon. Ces résultats contrastent avec les résultats de l'étude sur l'évaluation des préférences et des niveaux de satisfaction concernant les méthodes de paiement numérique menée en Inde par Deepa *et al*. en 2021 où 67% des participants étaient de sexe féminin et 70% célibataires [18]. La moyenne d'âge était de 26,96 ± 5 ans, ce qui est inférieur à celle trouvée en Inde en 2020 par Joshi *et al*. dans leur étude de faisabilité et d'efficacité d'un système de paiement numérique en ligne et de suivi des performances avec 35,51 ± 7 ans [10]. Près des deux tiers soit 61,72% des agents de santé avaient un revenu mensuel inférieur à cinquante mille francs CFA. Cela est en phase avec le faible niveau socio-économique des populations du département de Koumpentoum [13]. Le paiement numérique pourrait constituer une opportunité d'inclusion financière et de relance économique [8,9,14]. Ces variations dans les caractéristiques démographiques peuvent être attribuées aux contextes très différents des deux régions étudiées, et mettent en lumière l'importance de prendre en compte les facteurs culturels, économiques et sociaux dans l'interprétation des résultats.

La proportion d'agents utilisant le paiement numérique dans leur routine était de 77,36%. Cette proportion était supérieure à celle rapportée au Texas en 2017 par Johnson *et al*. dans leur étude sur les limitations à l'adoption rapide des services de paiement numérique en vue de comprendre l'impact du risque sur la protection de la vie privée avec 45% d'utilisateurs en routine [19]. De plus, nous avons noté une forte acceptabilité du paiement numérique dans d'autres aspects de la vie quotidienne atteignant 96,65%, ainsi que dans le secteur de la santé avec une acceptabilité de 86,6%. L'acceptabilité du paiement mobile était très élevée avec 91,5%. La majorité des agents de santé ayant été payés par voie numérique dans le secteur de la santé étaient satisfaits, avec un taux de satisfaction de 95,58%. Cette proportion très élevée d'utilisateurs satisfaits a également été rapportée au Nigeria en 2014 par Tella *et al*. dans l'étude de cas à l'université d'Ilorin portant sur les prédicteurs de la satisfaction des utilisateurs à l'égard du système de paiement électronique [11]. Ces résultats suggèrent que le paiement numérique, en particulier le paiement mobile est bien accepté par les agents de santé dans notre étude. La forte acceptabilité du paiement numérique dans le secteur de la santé pourrait encourager les autorités de santé à promouvoir davantage son utilisation, ce qui pourrait améliorer l'efficacité et la transparence des transactions financières dans le système de santé.

De plus, en réduisant la paperasserie et la gestion de l'argent liquide, le paiement numérique pourrait simplifier la gestion financière et réduire les risques de fraude, ce qui est bénéfique pour l'intégrité du système de santé. Ces conclusions positives peuvent contribuer à souligner le potentiel du paiement numérique dans le secteur de la santé, ce qui pourrait être bénéfique pour la planification et la gestion des ressources humaines pour le renforcement du système de santé sénégalais.

Notre étude avait révélé qu'une expérience professionnelle de moins de 5 ans était prédictive de l'acceptabilité contrairement à l'étude de Joshi *et al*. en Inde en 2020 [10]. Cela pourrait s'expliquer par le fait que les jeunes agents de santé auraient davantage tendance à utiliser les technologies numériques que leurs anciens. En effet, cette association entre le plaisir perçu et l'intention d'utilisation du paiement numérique a été relevé en Indonésie par l'étude de Nur *et al*. en 2021 [20] portant sur les facteurs influençant l'adoption de la méthode de paiement mobile et celle réalisée en Jordanie en 2018 par Alalwan *et al*. [21] qui examinait les facteurs influençant les intentions des clients jordaniens et l'adoption des services bancaires par Internet.

L'acceptabilité du paiement numérique dans la routine était aussi associée à son acceptabilité dans le domaine de la santé. Nous avions aussi noté une association entre l'attente de performances et l'acceptabilité du paiement mobile. Ce même constat a été aussi noté dans les études d'Alalwan *et al*. en Jordanie en 2018 [21], de Christine *et al*. [22] en 2018 en Indonésie dans l'évaluation du système de traitement des polices dans une compagnie d'assurance générale, d'Al-Okaily *et al*. en 2020 en Jordanie [15] et de Khalilzadeh *et al*. en 2017 aux Etats Unis [23] dans l'étude portant sur les facteurs liés à la sécurité du paiement mobile dans l'industrie de la restauration. Cela s'expliquerait par le fait que les technologies numériques sont souvent associées à la performance dans tous les secteurs de la vie économique [8,9]. Nous n'avions pas noté de lien entre la sécurité perçue et l'acceptabilité du paiement numérique contrairement Alalwan *et al*. [21] en 2018 en Jordanie, Khalilzadeh *et al*. en 2017 aux Etats Unis [23], Shin *et al*. en 2007 en Chine sur l'application des modèles de la technologie numérique [24] et à Shao *et al*. dans son étude sur les antécédents de la confiance et de l'intention de continuité dans les plateformes de paiement mobile [25]. Nous n'avions pas noté de lien entre le sexe et l'acceptabilité du paiement mobile contrairement à Shao *et al*. en 2018 en Chine [25] qui a révélé l'effet modérateur du genre dans l'adoption du paiement mobile. En effet, la mobilité et la réputation aurait une influence plus forte sur la confiance des hommes, tandis que la personnalisation et la sécurité ont une influence plus forte sur la confiance des femmes [25].

Notre étude n'a pas révélé des liens entre la valeur du prix et l'acceptabilité du paiement mobile contrairement à Al-Okaily *et al*. en 2020 [15] et par Alalwan *et al*. en 2018 [21] en Jordanie. Selon ces auteurs la sécurité perçue et la valeur du prix sont des déterminants importants de l'adoption des technologies médicales. Nous n'avons pas retrouvé de lien entre la vitesse perçue et l'acceptabilité du paiement numérique contrairement à Tella *et al*. au Nigeria en 2014 [11], Shin *et al*. en 2007 [24] en Chine, Al-Okaily *et al*. en 2020 en Jordanie [15], Shao *et al*. en 2018 en Chine [25], Khalilzadeh *et al*. en 2017 aux Etats Unis [23] et Johnson *et al*. en Inde en 2020 [19]. Notre recherche n'a pas révélé de lien entre la facilité perçue et l'acceptabilité. Par contre Sigar en 2016 en Indonésie a noté une influence positive significative entre la facilité d'utilisation et l'intention d'adopter le paiement numérique [26]. De même selon l'étude d'Alalwan *et al*. réalisée en Jordanie en 2018, l'espérance d'effort influence négativement l'adoption du paiement numérique [21].

Ces résultats mettent en lumière la complexité des déterminants de l'acceptabilité du paiement numérique dans le contexte spécifique des agents de santé. Ils fournissent des indications importantes pour orienter les initiatives visant à promouvoir l'adoption réussie du paiement numérique dans le secteur de la santé. Il serait alors important de sensibiliser et de former les acteurs du système de santé à l'utilisation correcte des technologies numériques et des plateformes de paiement pour maximiser les avantages du paiement numérique dans le secteur de la santé. Enfin, la collaboration avec les partenaires du secteur privé pourrait permettre le développement de solutions de paiement numérique spécifiques au secteur de la santé, en veillant à ce qu'elles soient conviviales et accessibles pour tous les acteurs du système de santé.

**Limites de l'étude:** nous n'avons pas pu enquêter auprès de tous les agents de santé. Cela pourrait affecter la généralisabilité des résultats à l'ensemble de la population des agents de santé. La rareté des études sur le paiement numérique en santé a limité notre capacité à comparer et discuter certains résultats avec des travaux antérieurs. Cette absence de données de référence a rendu difficile l'interprétation de certains aspects de notre étude.

## Conclusion

La numérisation des paiements pour les agents de santé pourrait non seulement améliorer leur bien-être, mais également celui des patients et les performances globales du système de santé. Notre étude, pionnière au Sénégal, se concentre spécifiquement sur les agents de santé. Nous avons constaté une acceptabilité très élevée, atteignant 91,5%, avec des facteurs favorisants incluant une expérience professionnelle de moins de 5 ans, une expérience positive du paiement mobile, une utilisation régulière du paiement mobile dans la vie quotidienne, ainsi qu'une attente de performances élevées. Sur la base de ces résultats, nous recommandons aux décideurs d'adopter le paiement numérique dans les initiatives de santé, en mettant l'accent sur la sensibilisation à ses avantages, notamment en termes de rapidité, de sécurité, d'anonymat et d'efficacité en matière de gestion du temps. Il est également crucial de réduire les frais d'utilisation, d'étendre la couverture du réseau téléphonique et Internet, ainsi que d'améliorer la gestion des réclamations pour assurer une transition fluide vers ce mode de paiement et en maximiser les avantages potentiels.

### Etat des connaissances sur le sujet

- La numérisation des paiements a été accélérée par la pandémie de COVID-19, avec des avantages perçus liés à la commodité et la rapidité;
- En Afrique, le paiement mobile se développe, mais une majorité de travailleurs reçoivent encore leur salaire en espèces;
- Facteurs influençant l'acceptabilité: des études antérieures ont montré que la commodité, la sécurité, la rapidité, et la confiance sont des facteurs clés; Ces éléments mettent en lumière l'importance des caractéristiques sociales et économiques locales dans l'adoption de nouvelles technologies de paiement.

### Contribution de notre étude à la connaissance

- Facteurs culturels et comportementaux: exploration des barrières et moteurs culturels spécifiques à la région concernant l'acceptabilité des paiements numériques;
- Mise en perspective avec d'autres régions pour comprendre les différences et similitudes dans l'adoption;
- Contribution de données originales collectées localement, enrichissant la littérature existante.

## Remerciements

Nous remercions l'équipe cadre de district, les infirmiers chef de postes, tous les agents du Koumpentoum et le projet *Digital Health Payment Initiative et Research (DHPI-R)*.
